# Unraveling the Gut Microbiome–Diet Connection: Exploring the Impact of Digital Precision and Personalized Nutrition on Microbiota Composition and Host Physiology

**DOI:** 10.3390/nu15183931

**Published:** 2023-09-11

**Authors:** Giada Bianchetti, Flavio De Maio, Alessio Abeltino, Cassandra Serantoni, Alessia Riente, Giulia Santarelli, Maurizio Sanguinetti, Giovanni Delogu, Roberta Martinoli, Silvia Barbaresi, Marco De Spirito, Giuseppe Maulucci

**Affiliations:** 1 Department of Neuroscience, Biophysics Sections, Università Cattolica del Sacro Cuore, Largo Francesco Vito, 1, 00168 Rome, Italy; giada.bianchetti@unicatt.it (G.B.); alessio.abeltino@unicatt.it (A.A.); cassandra.serantoni@unicatt.it (C.S.); alessia.riente@unicatt.it (A.R.); marco.despirito@unicatt.it (M.D.S.); 2 Fondazione Policlinico Universitario “A. Gemelli” IRCCS, 00168 Rome, Italy; 3 Dipartimento di Scienze di Laboratorio e Infettivologiche, Fondazione Policlinico Universitario “A. Gemelli” IRCCS, 00168 Rome, Italy; flavio.demaio@unicatt.it (F.D.M.); giulia.santarelli@unicatt.it (G.S.); maurizio.sanguinetti@unicatt.it (M.S.); 4 Dipartimento di Scienze Biotecnologiche di Base, Cliniche Intensivologiche e Perioperatorie, Sezione di Microbiologia, Università Cattolica del Sacro Cuore, 00168 Rome, Italy; giovanni.delogu@unicatt.it; 5 Mater Olbia Hospital, 07026 Olbia, Italy; 6 Società Italiana di Medicina Estetica, 00195 Rome, Italy; dott.roberta.martinoli@gmail.com; 7 Department of Movement and Sports Sciences, Faculty of Medicine and Health Sciences, Watersportlaan 2, Ghent University, 9000 Ghent, Belgium; silvia.barbaresi@ugent.be

**Keywords:** precision nutrition, gut microbiome, dietary intervention, longitudinal studies, nutrigenomics, personalized medicine

## Abstract

The human gut microbiome, an intricate ecosystem housing trillions of microorganisms within the gastrointestinal tract, holds significant importance in human health and the development of diseases. Recent advances in technology have allowed for an in-depth exploration of the gut microbiome, shedding light on its composition and functions. Of particular interest is the role of diet in shaping the gut microbiome, influencing its diversity, population size, and metabolic functions. Precision nutrition, a personalized approach based on individual characteristics, has shown promise in directly impacting the composition of the gut microbiome. However, to fully understand the long-term effects of specific diets and food components on the gut microbiome and to identify the variations between individuals, longitudinal studies are crucial. Additionally, precise methods for collecting dietary data, alongside the application of machine learning techniques, hold immense potential in comprehending the gut microbiome’s response to diet and providing tailored lifestyle recommendations. In this study, we investigated the complex mechanisms that govern the diverse impacts of nutrients and specific foods on the equilibrium and functioning of the individual gut microbiome of seven volunteers (four females and three males) with an average age of 40.9 ± 10.3 years, aiming at identifying potential therapeutic targets, thus making valuable contributions to the field of personalized nutrition. These findings have the potential to revolutionize the development of highly effective strategies that are tailored to individual requirements for the management and treatment of various diseases.

## 1. Introduction

The human gut microbiome, consisting of trillions of microorganisms residing in the gastrointestinal tract, has emerged as a complex ecosystem that plays a crucial role in human health and disease [1]. It comprises a diverse community of bacteria, archaea, viruses, fungi, and other microorganisms, collectively referred to as the gut microbiota. Recent advances in high-throughput sequencing technologies have enabled a comprehensive exploration of the gut microbiome and provided insights into its composition and functions [2].

The gut microbiome has garnered significant attention due to its profound impact on various aspects of human physiology, including nutrient metabolism, immune system development and regulation, host defense against pathogens, and even neurological functions [3,4]. Among the factors influencing the gut microbiome, diet has emerged as a key modulator of its composition and function [5]. The relationship between the gut microbiome and food is an ongoing and reciprocating interaction that has generated substantial interest. However, the precise mechanisms linking the gut microbiome and diet remain elusive. It is well-established that the composition of an individual’s diet exerts a profound influence on the diversity and abundance of gut microbes [6]. Indeed, studies have shown that different dietary patterns, such as high-fiber, plant-based diets versus Western-style, high-fat diets, can result in distinct microbial communities in the gut [7]. These dietary variations can modulate the production of microbial metabolites, such as short-chain fatty acids (SCFAs) [8], which have been linked to numerous health benefits, including improved metabolic health and reduced risk of chronic diseases [9]. Conversely, the gut microbiome possesses the capacity to shape how the host digests, absorbs and metabolizes food components.

This intricate interplay between the gut microbiome and dietary factors holds substantial implications for human health and disease, also being implicated in the development of obesity [10], type 2 diabetes [11], inflammatory bowel diseases [12], and other disorders [13]. In this perspective, specific dietary supplements, such as prebiotics and probiotics, have been investigated for their ability to shape the gut microbiota composition [14]. Prebiotics, including dietary fibers, serve as substrates for beneficial gut bacteria, promoting their growth and activity [15]. Probiotics, on the other hand, are live microorganisms that confer health benefits when consumed in adequate amounts [16].

As the field of microbiome research continues to advance, innovative techniques and approaches, including metagenomics, metabolomics, and computational modeling, are being employed to unravel the intricate relationship between the gut microbiome and food [17]. These studies aim to provide a deeper understanding of the mechanisms by which the gut microbiome influences host physiology and to identify potential therapeutic targets for personalized nutrition and disease management [18]. Indeed, since recent research has shown that personalized nutrition interventions have the potential to directly influence and modify the composition of the gut microbiome [19], precision nutrition is emerging as a tool that aims to provide personalized dietary recommendations based on an individual’s unique characteristics [20].

To this aim, it is crucial to collect real-time data on multiple factors that influence an individual’s response to diet, gather information on their current dietary habits, analyze their genetic makeup, and understand epigenetic modifications that can impact gene expression. However, one of the challenges in implementing precision nutrition is the high degree of inter-individual variability in dietary responses [21]. Indeed, different people may exhibit diverse physiological and metabolic characteristics that influence how their bodies process and respond to specific foods and nutrients. This means that to advance precision nutrition interventions targeting the gut microbiome, it is essential to conduct longitudinal studies that can help uncover the long-term effects of specific diets or food components on the gut microbiome composition, diversity, and function, also allowing for the identification of inter-individual variability and the factors that influence an individual’s response to dietary interventions [22]. It is furthermore important to acknowledge that the design of effective personalized nutrition interventions targeting the gut microbiome requires a deeper understanding of microbial ecology, host-microbe interactions, and the complex interplay between various dietary components and the microbiome, making this kind of study crucial to expand the knowledge base and refine the design of precision nutrition interventions [23].

Moreover, given the availability of advanced sequencing technologies, it becomes increasingly important to complement microbiome data with robust and precise dietary data. Many studies rely on methods such as food frequency questionnaires (FFQs), self-administered single-day food records, or 24 h dietary recalls to assess dietary intake. However, these methods have limitations and may not capture the intricate relationships between diet and the gut microbiome [24,25]. To address this, there is a need for improved methods to assess and collect dietary data for microbiome studies according to food preferences rather than relying solely on nutrient intake [26]. Additionally, the application of machine learning (ML) approaches in this field holds great potential. ML has been widely used in biomedical research and can diagnose or predict the risk of various health conditions, including, among others, cancer [27] and metabolic impairments [28]. In sports science, ML approaches can enhance research on the connection between the microbiome and exercise [29]. By predicting an athlete’s exercise responsiveness and identifying the key factors influencing their physiology, ML models can provide personalized lifestyle recommendations to optimize an athlete’s microbiota and improve their overall health. However, it is important to ensure the careful design of data collection processes, use quantitative and objective target variables, and prioritize interpretable ML models to enhance the understanding and interpretation of results. In this perspective, collaborations across disciplines are crucial to address challenges and establish a common ground for knowledge transfer.

Indeed, the high degree of inter-individual variability necessitates robust analytical approaches that can effectively account for this variability and assess the effectiveness of dietary interventions. One such method is the paired *t*-test, which is particularly valuable in longitudinal studies where participants serve as their own controls. By comparing measurements within individuals before and after an intervention, the paired *t*-test enables the identification of significant changes within individuals while minimizing the impact of inter-individual variability. This approach allows evaluation of the specific effects of dietary interventions on the gut microbiome composition, diversity, and function, providing valuable insights into the most effective strategies for personalized nutrition. By using this statistical method, it is possible to better discern the true impact of interventions on the gut microbiome and make informed decisions regarding precision nutrition recommendations tailored to an individual’s unique needs and responses.

In this context, our study focused on personalized and precision nutrition, aiming to investigate the influence of diet on the microbiota of a specific group of individuals. Through a tailored nutritional intervention, we sought to gain a comprehensive understanding of how individual dietary factors impact the composition and function of the gut microbiota and how these changes relate to various aspects of host physiology, including anthropometric and physiological parameters. By investigating and unraveling these complex underlying mechanisms, our research sought to shed light on the intricate and multifaceted individual impact, discerning not only the differential effects of various nutrients but also the influence of specific foods on the balance and functionality of the microbiome. Our approach involves meticulously tracking participants’ dietary intake and correlating it with changes in their physiological markers. By adopting this indirect strategy, we aim to establish a robust cause-and-effect relationship between dietary interventions and physiological outcomes. We believe that this methodology not only leverages the expertise of nutritionists but also provides a systematic framework for assessing the impact of dietary changes on health-related variables. In doing so, we aim to create a library of interventions that are linked to specific outcomes, enhancing the potential for broader applicability beyond the scope of specialized nutritional knowledge. Through this in-depth exploration, we aimed to make significant contributions to the identification and elucidation of potential therapeutic targets, thereby paving the way for the development of highly effective personalized nutrition strategies tailored to the unique needs of individuals and enabling the effective management and treatment of diverse diseases.

## 2. Materials and Methods

### 2.1. Study Population

**Figure 1:**
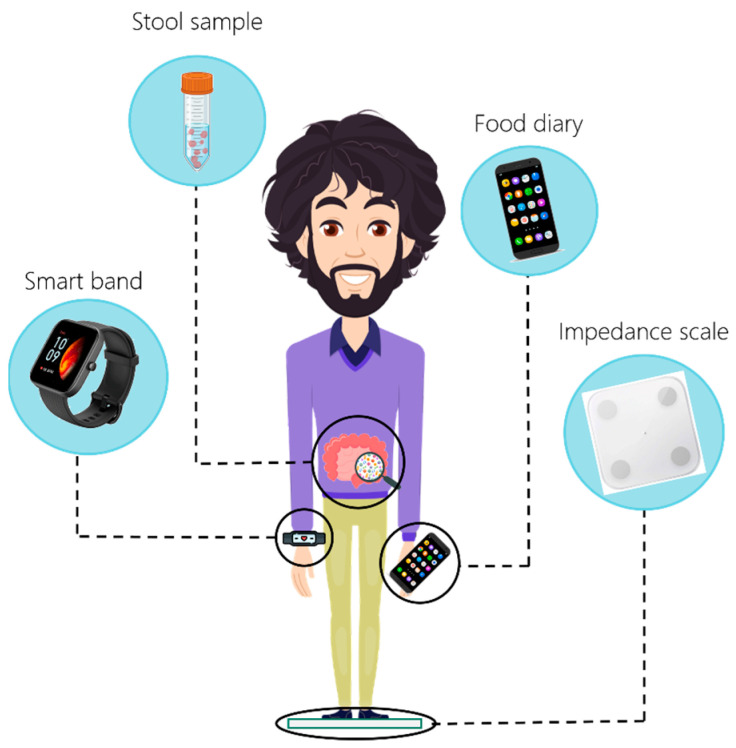
**Data acquisition.** Representation of the wearables, devices, and techniques used to extract the comprehensive data integrated into the personalized approach from the participants. An impedance scale allows evaluating anthropometric parameters; the smart band furnishes information regarding physiological parameters and physical activity data; the web app ArMOnIA ensures a detailed compilation of food diary; the collection of a stool sample is required for microbiome analysis.

In this single-arm, uncontrolled-pilot prospective study, 7 volunteers (4 females = 57%, and 3 males = 43%, age = 40.9 ± 10.3 years, Body Mass Index (BMI) = 23.2 ± 2.9 kg/m^2^) were recruited from our lab staff and asked to self-monitor their weight, diet, and activities as previously described in [19] and represented in Figure 1, between March and July 2022. The involved subjects were deemed “recruitable” due to their absence of antibiotic treatments or probiotic cycles in the weeks prior to the study. The study was conducted in accordance with the Declaration of Helsinki and approved by the Ethics Committee of Policlinico Universitario “A. Gemelli” IRCCS for studies involving humans (ID: 5407—Prot. 2336/23). Written informed consent was obtained from each subject involved in the study.

### 2.2. Timeline

**Figure 2:**
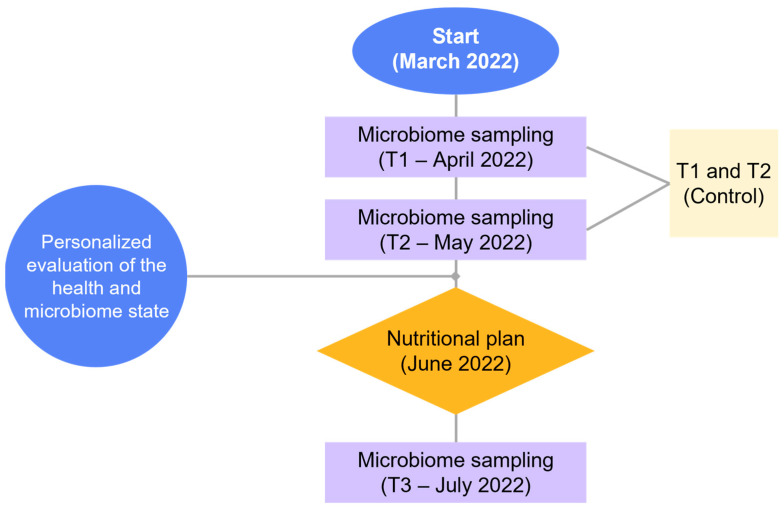
**Study timeline.** Schematic representation of the study timeline. Purple rectangles show the microbiome sampling, the orange diamond represents the intervention, while in the blue circles, the starting point and the intermediate evaluation of the results are provided.

In Figure 2, a schematic representation of the study timeline is reported. The study was conducted starting in March 2022. Samples for nutrigenomics were collected in April 2022. The research, as shown in the purple rectangles, involved two microbiome sampling time points, namely T1 (April 2022) and T2 (May 2022), with T2 conducted shortly after T1. The T1 data served as the initial data point, while the T2 sampling aimed to provide additional insight and verify the reliability of the initial findings (see Section S1, Supplementary Materials). Personalized evaluation of the participant’s health and microbiome state was performed by averaging the data from T1 and T2, which served as the control group (yellow square). This control value will be referred to as T_CTRL_ from now on. In June 2022, a tailored nutritional plan was implemented for each participant (orange diamond). Subsequently, another round of microbiome sampling indicated as T3 and referred to as T_DIET_, was carried out in July 2022 (one-month duration of the nutritional plan) to assess the impact of the personalized nutritional intervention. The selection of one month for our nutritional plan was based on the highly dynamic nature of the microbiome, which can respond relatively quickly to dietary alterations characterized by great adaptability. Indeed, it can adjust to different dietary patterns, with specific microbial populations flourishing or declining based on the presence or absence of certain nutrients. This is substantiated by studies that have shown significant changes in gut microbiome composition following dietary interventions typically lasting a few weeks to a month [30,31]. Moreover, while it is true that substantial shifts in the overall microbial composition may require a longer duration, intermediary changes like variations in specific microbial metabolites or short-chain fatty acids can be observed within this time interval [32], making it reasonable to detect any changes occurring as a result of dietary modifications.

### 2.3. Sample Collection Procedure for Nutrigenomics

For the nutrigenomics analysis, a saliva sample was required. The participants were instructed to follow a supervised collection procedure to ensure the accuracy and integrity of the DNA sample. The collection process involved refraining from eating, drinking, smoking, or performing any oral hygiene activities for at least 30 min prior to collection. Lipstick was required to be removed to prevent external contamination. Using a sterile swab, participants vigorously rubbed the inside of their cheeks and gums for approximately 1 min, following specific circular motions. After collection, the swab was left to dry in the air for approximately 1 min, ensuring that it did not come into contact with any surfaces, before being carefully placed back into the provided collection tube. Each participant’s sample was assigned a unique serial number to maintain anonymity throughout the analysis process, which was performed by iDNA Genomics (Kifisia, Greece).

To explore genes and genetic variations related to nutrition, multiple sources, including scientific research papers and genetic databases, have been consulted, including, for example, NutriGenomeDB [33]. After the selection of genes, an extensive assessment of the following genes (Table 1) was performed, encompassing a comprehensive evaluation of their specific genotypes for each participant. The list here constitutes a standard panel and was sent to the industry partner iDNA Genomics, who performed the quantification.

### 2.4. Sample Collection Procedure and Analysis of Microbiome

Fecal samples were collected using DANASTOOL tubes and stored at room temperature until DNA extraction (for a maximum of 7 days). For each sample, DNA extraction was performed in a strictly controlled level-2 biological safety workplace. In keeping with a previously described protocol [34], we used 200 µL of suspended fecal sample in hexadecyltrimethylammonium bromide (CTAB) buffer to extract bacterial DNA with the DANAGENE MICROBIOME Fecal DNA kit (Danagen-Bioted, Barcelona, Spain). DNA was eluted in 200 µL of pre-heated nuclease-free water and stored at −20 °C until sequencing processing. DNA concentration was assessed using the Qubit 4 fluorometer (Thermo Fisher Scientific, Waltham, MA, USA) and ds DNA High sensitivity assay (Thermo Fisher Scientific) according to manufacturer’s procedures. V3–V4 and V6 hypervariable regions of the 16S rRNA gene were amplified by using Microbiota solution B (Arrow diagnostics) and prepared for the paired ends sequencing (2 × 250 bp, v2 chemistry, Illumina, San Diego, CA, USA) on the Illumina MiSeq instrument (Illumina) [35,36]. Raw data were firstly processed using MicrobAt suite software v1.2.1 provided by SmartSeq bioinformatic and analyzed using R v4.0.2 (https://www.rstudio.com/, accessed on 7 September 2023) and phyloseq package for downstream analyses in-house analysis pipeline [37]. Unassigned amplicon sequence variants (ASVs) and taxonomic and prevalence-based pre-filtering (to exclude ASVs with small mean and trivially large variation) were applied. Samples were normalized to minimize the effect of the sequencing depth differences, and alpha diversity was calculated. Alpha diversity metrics were computed as Shannon diversity index and Pielou’s evenness. Beta diversity was evaluated using Bray-Curtis graphically represented as principal coordinate analysis (PCoA). Significance between microbial community composition was obtained using the adonis function included in the vegan package, which performs permutational multivariate analysis of variance (PERMANOVA). Before relative abundances were measured, phyla representing less than 1% (Fusobacteria, Lentisphaerae, Synergistetes, Tenericutes) were grouped and indicated as “<1% phyla”. Relative abundances were measured at phylum, genus, and species levels, and statistical significance was assessed by the Wilcoxon signed rank test.

### 2.5. Formulation of the Personalized Nutritional Plan

After the meticulous collection and analysis of data from the two microbiome control samples, as well as comprehensive assessments of anthropometric measurements, physiological parameters, genomic information, and physical activity levels, a highly personalized nutritional plan was developed. This intricate process involved two experienced nutritionists who collaborated closely, utilizing the cutting-edge software known as “Terapia Alimentare”, which is based on the methodology developed by Dietosystem, a division of DS MediGroup S.p.A. (Terapia Alimentare Dietosystem^®^ v19.00, DS-Medica, http://www.dsmedica.info, accessed on 7 September 2023). The software provided valuable guidance, evidence-based recommendations, and data analysis tools that supported the nutritionists throughout the process. These tools leverage comprehensive nutritional databases, which allow them to calculate the nutritional content of a broad spectrum of foods and recipes. Such databases encapsulate details about macronutrients and micronutrients, in addition to aspects like dietary fiber content and the glycemic index of foods. In conjunction with this, these applications also incorporate established dietary guidelines from trusted organizations like the World Health Organization [38] or the Food and the European Food Safety Authority [39], as well as the specific national dietary guidelines of various countries. This information provides a roadmap for the recommended intake of different nutrients for various demographic groups, which the software utilizes to generate well-rounded meal plans. By harnessing the combined power of the nutritionists’ knowledge and the software’s comprehensive features, a truly tailored and effective nutritional plan was created for each participant. For a more comprehensive understanding of how our dietary interventions were tailored to each participant based on their genetic and microbiome profiles, see Supplementary Materials (Section S2) [40,41,42,43,44]. In particular, Table S2 provides a detailed account of the individualized dietary recommendations, including specific genetic variations (SNPs) that were taken into consideration by our expert nutritionists. Additionally, it outlines the corresponding nutritional guidance provided to optimize the growth and activity of beneficial bacteria while minimizing the proliferation of potentially harmful ones, serving as a valuable resource for those seeking a deeper insight into the personalized nature of our dietary interventions.

### 2.6. Data Organization and Analysis

To evaluate the diet effect, we assessed a comprehensive set of variables collected through the home-developed web app ArmOnIA (https://www.apparmonia.com/, accessed on 7 September 2023) [45,46,47]. The collected variables can be divided into 3 main groups: anthropometric and physiological; microbiome; and nutritional parameters. Age, weight, metabolic rate in kilocalories, body mass index (BMI), percentage of body fat, muscle mass, bone mass, percentage of body water, daily physical activities, resting heart rate, average heart rate, duration of deep sleep, duration of shallow sleep, and rapid eye movement (REM) sleep were among the physiological parameters measured.

To gain insights into the microbial composition, microbiome analysis was assessed through key parameters: richness, a measure of the number of unique taxa present in a sample; Pielou’s evenness index, to assess the distribution of abundances among different taxa; Shannon diversity index, to evaluate the overall diversity and species richness within the microbiome. In addition to these diversity metrics, we examined the relative abundances of phyla, genera, and species in the microbial community.

Regarding the nutritional area, we examined specific nutritional parameters, including the fraction of protein, carbohydrates, fats, and dietary fibers, intake of kilocalories, and the thermal effect of food (TEF), which refers to the energy expenditure occurring to digest, absorb, and metabolize the nutrients from the food. These parameters provided insights into the macronutrient composition and energy content of the food categories. Furthermore, we analyzed the levels of essential minerals such as calcium, iron, magnesium, potassium, phosphorus, sodium, zinc, copper, and manganese. The foods recorded in the web app were categorized into 141 classes starting from the classification system established by the Food and Agriculture Organization (FAO) and World Health Organization (WHO) [48]. Building upon this classification, we further created additional groups by combining specific variables. As a result, we defined distinct categories such as white fish, oily fish, low-fat cheeses, high-fat cheeses, fresh cheeses, medium-aged cheeses, and long-aged cheeses. These refined categories allowed for a more detailed analysis and understanding of the nutritional characteristics and composition of different food items. By grouping the foods based on these additional categories, we were able to uncover valuable insights and patterns in relation to their nutritional profiles.

For each of these variables and each participant, the mean values related to the time interval between two subsequent microbiome samples were quantified and used for the comparative analysis and the evaluation of the personalized nutritional plan effect. In particular, the participant’s health and microbiome state was evaluated by comparing the pre-intervention data (T_CTRL_) and the post-intervention data (T_DIET_).

### 2.7. Statistics

For each of the variables previously described, a paired *t*-test was performed using Python 3.10 (https://www.python.org/, accessed on 12 June 2023). The statistical analysis was conducted using the libraries pandas (https://pypi.org/project/pandas/, accessed on 12 June 2023), numpy (https://pypi.org/project/numpy/, accessed on 12 June 2023), and scipy (https://pypi.org/project/scipy/, accessed on 12 June 2023). The paired *t*-test is specifically designed for comparing two related samples, making it suitable for evaluating the significance of differences between the initial measurements (before the diet, denoted as T_CTRL_) and the final measurements (after the diet, denoted as T_DIET_). FDR correction was applied to the obtained *p*-values. Beta diversity was investigated using Bray-Curtis distance and represented by principal coordinates analysis (PCoA). Statistical differences were assessed by permutational multivariate analysis of variance (PERMANOVA) test.

## 3. Results

### 3.1. Precision Nutrition

The precision nutrition approach employed in this study represented a cutting-edge methodology that goes beyond traditional dietary recommendations. By incorporating a holistic analysis of participants’ genetic profile, obtained by nutrigenomics analysis, microbiome composition, and various physiological parameters, including anthropometric measurements and physical activity levels (as outlined in Table S2 in the Supplementary Materials), we gained a comprehensive understanding of each individual’s unique health characteristics.

Leveraging this wealth of information, our nutritionists devised personalized plans tailored to the specific needs and goals of each patient, whose main characteristics are reported in Table 2.

Table 2 presented data on the participants’ age, diet type, and macronutrient percentages before the diet (% Macronutrient Intake) and provided by the personalized nutritional plan (% Macronutrients Diet-provided). The participants followed different nutritional approaches, including the Mediterranean diet and a Ketogenic/Low-Carbohydrate diet.

In the Mediterranean diet group, participants WL010114, WL010112, WL010111, WL010107, WL010106, and WL010105 adhered to a diet characterized by a moderate carbohydrate intake ranging from 42.2% to 47.9% of total calories, protein intake varied between 21.7% and 24.4%, while fat intake ranged from 29.5% to 34.9% of total calories. Notably, participants WL010112, WL010111, and WL010105 within the Mediterranean diet group followed a variation of the diet with a high fish intake. Participant WL01008 followed a different dietary approach known as the Ketogenic or Low-Carbohydrate diet. This participant consumed a significantly lower proportion of carbohydrates, accounting for 36.9% of total calories, while protein intake was relatively high at 33.3% of total calories. Fat intake for participant RC008 was 29.8% of total calories.

An innovative aspect of the diet formulation was the incorporation of the genomic profile and microbiome of each participant. The integration of genomic data, specifically genetic variations such as single nucleotide polymorphisms (SNPs), allowed us to gain insights into potential gene expression patterns that may influence an individual’s metabolism and response to dietary components. These genetic variations can serve as proxies for understanding how certain genes may be expressed or regulated in a person’s body. In this sense, to prescribe diets to the participants, the nutritionists applied a two-pronged approach, drawing insights from both scientific literature and their extensive expertise in the field of nutrigenomics. To elucidate how the genetic information was translated into dietary recommendations, we have provided a detailed account in Supplementary Table S2, which serves as a comprehensive reference that highlights key points of the interventions for each participant, including specific genetic variations and their implications. It is important to highlight that these comments and considerations encompass a wide range of factors, such as genetic predispositions to lactose intolerance, metabolic responses to certain nutrients, and responses to dietary components linked to weight management and sensitivities. By taking into account the individual information about specific genetic variations together with microbial composition, the nutritionists gained valuable insights into the specific bacterial species present. This data-driven approach enabled diet customization to optimize the growth and activity of beneficial bacteria while minimizing the proliferation of potentially harmful ones. By integrating both genomic and microbiome data into the nutrition planning process, the dietary interventions were tailored to meet the subjects’ nutritional requirements while also fostering a favorable microbial ecosystem. This groundbreaking methodology, rooted in precision nutrition, underscores the intricate interplay between nutrition and the gut microbiome and, by addressing the intricacies of subjects’ genetic predispositions and considering their individual metabolic responses, highlights the effectiveness of this integrative approach in revolutionizing the field of nutrition, paving the way for precise and targeted dietary strategies.

### 3.2. Changes in Food Intake

One month after the administration of the personalized nutritional plan to the subjects involved in the study, another microbiome sampling was performed to evaluate the individual response to the diet. First, we investigated changes in food intake occurring after the intervention. In Table 3, the food categories that significantly changed after nutritional intervention among the 141 identified and previously introduced (see Section 2.6) are reported.

Table 3 presented data on changes in food items between two time points, T_CTRL_ (baseline) and T_DIET_ (final), in the study participants. The mean values and standard deviations (SD) were provided for each variable. In particular, it was interesting to observe a significant decrease in the mean consumption of cereal bars (from 13.1 ± 13.0 g at T_CTRL_ to 0.0 ± 0.0 g at T_DIET_, *p*-value = 0.05) and chocolate, from 18.7 ± 18.8 g at T_CTRL_ to 0.6 ± 1.7 g at T_DIET_, with *p*-value = 0.05.

In contrast, the mean consumption of ice cream increased significantly from 16.8 ± 23.1 g at T_CTRL_ to 60.7 ± 34.4 g at T_DIET_ (*p*-value = 0.05), together with the intake of Parmesan cheese, rising from 7.7 ± 4.4 g at T_CTRL_ to 24.2 ± 6.1 g at T_DIET_ (*p*-value = 0.05), and those of oily fish, almost doubling between T_CTRL_ (109.4 ± 93.5 g) and T_DIET_ (196.5 ± 131.5 g, *p*-value = 0.05).

### 3.3. Changes in Nutritional Variables

The second part of our analysis involved the evaluation of changes in the intake of several key nutrients among the study participants, whose results are reported in Table 4.

Table 4 presents data on various nutritional variables, including the total intake (in kilocalories), the fraction of macronutrients, and the mass of selected micronutrients before (T_CTRL_) and after (T_DIET_) the nutritional intervention. Interestingly, despite the total intake, as well as the fraction of macronutrients, carbohydrates, proteins, fats, and fibers, did not change after the nutritional intervention, we observed a significant increase in the intake of several micronutrients, including calcium, potassium, phosphorus, sodium, and zinc.

Adequate calcium intake is essential for maintaining strong bones and teeth, as well as for supporting various physiological processes in the body [49]. The participants showed a statistically significant increase in calcium intake (*p*-value = 0.05) after the dietary intervention, passing from 413 ± 121 mg at T_CTRL_ to 601 ± 251 mg at T_DIET_. Another important mineral is potassium, which plays a crucial role in maintaining proper electrolyte balance, supporting nerve function, and regulating blood pressure [50]. The significant increase in potassium intake, from 1476 ± 712 mg (T_CTRL_) to 1815 ± 615 mg (T_DIET_) (*p*-value = 0.05), indicates that the dietary intervention resulted in a noteworthy rise in the consumption of potassium-rich foods. In addition, the participants exhibited a significant increase in phosphorus intake (from 572 mg at T_CTRL_ to 709 mg at T_DIET_, *p*-value = 0.05), which is involved in numerous physiological processes, including energy metabolism, DNA synthesis, and bone health [51]. There was also a significant increase in sodium intake (*p*-value = 0.05), which rose from 1200 ± 717 mg to 1388 ± 760 mg after the diet. Sodium is an essential electrolyte that plays a role in maintaining fluid balance, nerve function, and muscle contraction. However, excessive sodium intake can contribute to high blood pressure, so it is important to monitor sodium intake within recommended limits [52]. Finally, a significant increase (*p*-value = 0.05) in zinc intake was observed, from 4 ± 2 mg at T_CTRL_ to 5 ± 2 mg at T_DIET_, an essential mineral involved in the enzymatic reaction, immune function, and DNA synthesis [53].

### 3.4. Effects of the Nutritional Plan on Anthropometric and Physiological Parameters of the Participants

To monitor the effects of the personalized diet on the anthropometric and physiological parameters of the participants, we further compared the mean values of weight, BMI, body composition, resting heart rate (RHR), and sleep quality before and after the nutritional intervention. The results, including the trend and the *p*-value obtained from the paired *t*-test, are reported in Table 5.

The first part of Table 5 provides an overview of the changes observed in various anthropometric parameters before and after the diet intervention. Participants experienced a significant decrease in BMI, from 23.1 ± 2.8 kg/m^2^ at T_CTRL_ to 22.4 ± 2.2 kg/m^2^ at T_DIET_ (*p*-value = 0.050), suggesting a positive trend towards improved body composition.

A decrease was also retrieved in basal metabolism, which represents the amount of energy expended by the body at rest, passing from 1327 ± 279 kcal to 1317 ± 249 kcal after the intervention. However, since this change was not statistically significant (*p*-value = 0.64), it may not be directly influenced by the diet intervention. In line with these observations, we observed a slight, although not significant, decrease in the percentage of body fat and bone mass, with a contextual increase in muscle mass and water percentage, representative of a shift towards a better state of health. The level of physical activity also did not change before and after the intervention.

In the second part of Table 5, we focused on the results observed on various physiological parameters before and after the diet intervention. Interestingly, there was a significant decrease in resting heart rate (RHR), with *p*-value = 0.05. This suggests that the diet may have had a positive impact on cardiovascular health, as a lower resting heart rate is generally associated with better heart function and fitness. While there were no significant changes observed in average heart rate, deep sleep, or REM sleep duration, there was a significant decrease in the duration of shallow sleep (*p*-value = 0.05), highlighting the complex relationship between diet and physiological parameters.

### 3.5. Effects of the Nutritional Plan on the GUT Microbiome

#### 3.5.1. Evaluation of the Stability of the Microbial Composition

**Figure 3:**
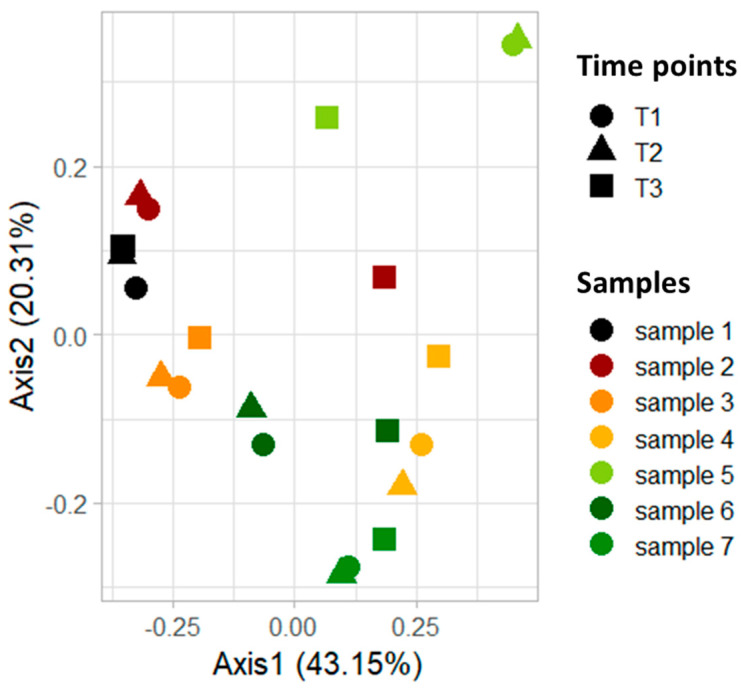
**Beta diversity analysis between T1, T2, and T3.** Beta diversity was investigated using *Bray–Curtis* distance and represented by principal coordinates analysis (PCoA). Statistical differences between the three groups (T1 and T2, as control groups, and T3 after diet) were assessed by Permutational Multivariate Analysis of Variance (PERMANOVA) test (*p* > 0.05). Different shapes represent different time points while varying colors are associated with the various studied samples.

To evaluate the stability and reliability of the individual’s gut microbiome before the nutritional intervention and the consequent effect of the personalized plan, the beta diversity, a measure reflecting the similarity or dissimilarity of the microbiome sampling, was evaluated using *Bray–Curtis* distance and represented by principal coordinates analysis (PCoA), see Figure 3. For the comparison of the two control time points before the nutritional intervention, see the Supplementary Materials, Figure S1.

The graph in Figure 3, which represents the two principal components on the x and y axis, highlights that the two points, T1 and T2 (circle and square, respectively), overlap for each sample, indicating no variation in the microbiota. Interestingly, looking at the PCoA representation of the beta diversity at T3, following the nutritional intervention (square point), a spatial shift in the microbial diversity can be observed, highlighting the effective impact of the personalized plan on the gut microbiome of different participants.

Statistical differences between the three groups (T1 and T2, as control groups, and T3 after diet) were assessed by permutational multivariate analysis of variance (PERMANOVA) test (see Supplementary Table S1), which relies on a distribution of data so that the only way to characterize the distribution is to have multiple samples. In this context, we can only graphically compare two points belonging to the same individual with a different spatial distribution (depending on unique or shared microbial features).

#### 3.5.2. Evaluation of the Changes in Composition after the Nutritional Intervention

To provide a quantitative evaluation of the changes induced in the gut composition by the nutritional intervention, we evaluated several variables related to diversity and microbial abundance. The results are reported in Table 6.

Table 6 displays the results of the analysis conducted on various microbiome parameters, including diversity, phyla, and species. Specifically, all the results for diversity and phyla were presented in the table, whereas for species, we only included those that exhibited statistically significant variations.

Richness, Pielou’s evenness, and Shannon diversity indicated different aspects of the microbial community structure, overall reflecting the gut microbiome diversity. Regarding richness, a significant increase was observed (*p*-value = 0.05), suggesting that the number of unique microbial species or taxa present in the gut increased from 409.7 ± 44.8 at T_CTRL_ to 528.1 ± 110.9 at T_DIET_, following the nutritional intervention. Pielou’s evenness, instead, referred to the distribution of microbial abundances, indicating how evenly the different species are represented. Although there was a slight increase in this parameter, rising from 0.627 ± 0.026 to 0.634 ± 0.029 between T_CTRL_ and T_DIET_, this change was not statistically significant. The last parameter, Shannon diversity, took into account both the number of species and their evenness in the community, so a higher value indicates a more diverse and balanced microbial ecosystem. In this case, we observed a significant increase (*p*-value = 0.05) following the administration of the personalized diet plan, from 3.76 ± 0.12 at T_CTRL_ to 3.96 ± 0.11 at T_DIET_. Overall, the results suggest that the intervention had a notable impact on the gut microbiome diversity, especially highlighting a greater number of unique species and a more diverse microbial community.

The second part of the table provided information on the relative abundance of different bacterial phyla in the gut microbiome and their corresponding statistical analysis results. In this case, we observed an increase in the relative abundance of Firmicutes (from 0.54 ± 0.14 at T_CTRL_ to 0.60 ± 0.15 at T_DIET_), Actinobacteria (from 0.07 ± 0.10 at T_CTRL_ to 0.08 ± 0.07 at T_DIET_), and Verrucomicrobia (from 0.02 ± 0.04 at T_CTRL_ to 0.04 ± 0.05 at T_DIET_), and a contextual decrease in the fraction of Bacteroidetes, passing from 0.27 ± 0.20 at T_CTRL_ to 0.21 ± 0.19 at T_DIET_, and Proteobacteria, with starting value of 0.08 ± 0.14 and final value of 0.06 ± 0.05 [54]. However, despite these variations, no significant changes were retrieved.

For this reason, we studied more in-depth, up to the level of bacterial species. The results are reported in the third part of the table. *Acinetobacter junii* showed a significant increase in abundance (*p*-value = 0.05), while *Alistipes finegoldii* exhibited a decrease in abundance (*p*-value = 0.05), indicating a significant reduction in its presence within the gut microbiome. Similarly, *Alistipes finegoldii* DSM 17242 showed a significant increase with a *p*-value of 0.05 (*), suggesting that this specific strain has become more abundant. *Bacteroides plebeius* displayed a decrease from (5.6 ± 5.5) × 10^−5^ at T_CTRL_ to (6.9 ± 6.8) × 10^−5^ at T_DIET_ (*p*-value = 0.05), indicating a significant reduction in its presence within the gut microbiome. Klebsiella sp. and its various strains, including Klebsiella sp. 8.1T, Klebsiella sp. XW111, Klebsiella sp. YSI6A and *Klebsiella variicola* all exhibited significant increases in abundance. The *p*-values ranged from 0.05 to 0.06, suggesting that these Klebsiella species have become more abundant within the gut microbiome. Similarly, *Lachnospiraceae bacterium* DJF RP14 and *Lachnospiraceae bacterium* DJF VP18k1 both showed significant increases in abundance, with values passing from (2.7 ± 5.7) × 10^−4^ and (1.5 ± 1.8) × 10^−4^ at T_CTRL_ to (3.9 ± 6.2) × 10^−4^ and (3.6 ± 3.2) × 10^−4^ at T_DIET_ (*p*-values of 0.05 and 0.03), respectively, together with *Lactobacillus crispatus*, which significantly increases from (4.2 ± 3.1) × 10^−5^ to (1.7 ± 1.6) × 10^−4^, following the nutritional intervention (*p*-value = 0.05). Conversely, *Roseburia faecis*, Roseburia sp. 11SE39, bacterium NLAE–zl–P167, Butyrate-producing bacterium PH07BW10, and Butyrate-producing bacterium SR1/5 all displayed significant decreases in abundance. The *p*-values indicate a significant reduction in the presence of these bacterial species within the gut microbiome.

## 4. Discussion

The present study employed a precision nutrition approach that incorporated a comprehensive analysis of participants’ genetic profiles, microbiome composition, and various physiological parameters to develop personalized dietary plans. The results demonstrated the efficacy of this approach in optimizing health characteristics and promoting a favorable microbial ecosystem. Indeed, the findings revealed significant changes in food intake, nutrient intake, anthropometric parameters, physiological parameters, and gut microbiome composition after the implementation of the personalized dietary intervention.

A first performed qualitative analysis showed consistent changes in food intake with an emphasis on increasing vegetables, legumes, and fresh fruit, which are rich in essential nutrients and dietary fiber. Omega-3 fatty acids from sources like fish and flaxseeds, known for their cardiovascular benefits [55], were also included with a contextual reduction in processed and refined foods in favor of whole grains and lean proteins. The incorporation of antioxidant-rich foods like lemon juice, cocoa powder, and green tea highlights the importance of combating oxidative stress and, together with other findings, supports the idea that a balanced and nutrient-rich diet, abundant in plant-based foods and healthy fats, can positively impact health and well-being [56].

Furthermore, while the summarized results shed light on the general trends and patterns observed across the study population, a longitudinal analysis allows us to assess changes within individuals over time. This approach enables us to capture personalized responses to dietary interventions, considering inter-individual variations (see Supplementary Materials, Section S3) and potential confounding factors. By employing paired *t*-tests, we can statistically compare the microbiome composition, nutrient profiles, and food intake of the seven individuals before and after dietary modifications, assessing changes longitudinally on each patient and using the individual’s baseline microbiota composition as the reference point, ultimately providing a more accurate assessment of the impact on their specific microbial ecosystem. This approach also enabled us to overcome the limitation related to the small number of subjects involved in our study.

The analysis of anthropometric parameters revealed positive trends towards improved body composition, with a significant reduction in BMI, suggesting weight loss. Moreover, there was a decrease in body fat and bone mass percentages, along with an increase in muscle mass and water percentage, indicating that the personalized dietary intervention may have contributed to favorable changes in participants’ body composition [57].

This positive trend is also confirmed by the observations in terms of physiological parameters, where a significant decrease in resting heart rate (RHR), generally associated with better cardiovascular health and fitness [58,59], is highlighted. Although no significant changes were observed in average heart rate, deep sleep, or REM sleep duration, there was a significant decrease in the duration of shallow sleep, suggesting that the personalized dietary intervention may have had a positive impact on cardiovascular health and sleep quality [60].

Interestingly, the evaluation of gut microbiome parameters revealed significant changes in diversity and species abundance, highlighting a significant increase in the number of unique microbial species (richness) and overall microbial diversity (Shannon diversity), which are representative of a more diverse and balanced microbial ecosystem. The importance of microbial diversity has been extensively studied in the field of microbiome research, with numerous scientific studies highlighting its significant role in maintaining various aspects of human health, including gut barrier function [61,62], immune system regulation [63,64], nutrient metabolism [65], mental health and brain function [66,67], and disease prevention [30].

Thanks to our in-depth analysis, we were able to monitor changes in the gut microbiome at the level of microbial species. In particular, in our study, we identified several microbes that exhibited significant changes in abundance following the dietary intervention, including *Acinetobacter junii,* which, according to recent studies, is involved in the metabolism of fats [68]. The observed higher consumption of high-fat foods, including ice cream, Parmesan cheese, and oily fish, can thus explain the correlation with diet. Similarly, *Alistipes finegoldii* DSM 17242, a bile-tolerant bacteria constituting a biomarker of the healthy gut [69], which is mostly associated with high-fat diets, showed a significant increase following the nutritional intervention, possibly being associated with the higher intake of Parmesan and oily fishes that, besides being high-fat foods, also elicit bile release [70,71].

It is worth noting that the observed significant increase in various Klebsiella species, such as *Klebsiella sp. 8.1T*, *sp. XW111*, *sp. YSI6A*, and *Klebsiella variicola* add an intriguing aspect to the discussion. The genus Klebsiella belongs to the family *Enterobacteriaceae* and can survive for extended periods in diverse environments, including dust, water, and animal or poultry feces [72,73]. This information raises the possibility that the higher consumption of fruits, vegetables, and fish in the Mediterranean diet model, which may be contaminated by water or soil, could contribute to the proliferation of Klebsiella species within the gut microbiome.

Furthermore, the observed increase in certain bacterial taxa, specifically *Lachnospiraceae* and *Lachnobacterium*, in response to the dietary intervention provides valuable insights into their potential metabolic roles and associations with specific food components. *Lachnospiraceae* are known for their involvement in carbohydrate catabolic pathways leading to the production of acetate and butyrate, as well as metabolic pathways of aromatic amino acids resulting in the release of beneficial compounds like indole-propionic acid, indole, phenol, and p-cresol. Previous studies have reported an increase in *Lachnospiraceae* abundances following a diet supplemented with omega-3 polyunsaturated fatty acids (PUFA), such as those found in oily fishes [74]. Additionally, the presence *of Lachnobacterium*, which has been positively associated with animal-derived nutrients and negatively correlated with vegetable-based diet patterns [75], suggests a potential connection to the consumption of animal-derived foods, including ice creams (often high in saturated fats due to the presence of milk), Parmesan cheese, and fish.

The higher intake of omega-3 and animal-derived foods can also be related to the observed decrease in the abundance of *Bacteroides Plebeius* and Roseburia species.

*Bacteroides plebeius*, known for its involvement in the metabolism of glycosaminoglycans, particularly dermatan sulfate (DS) and heparan sulfate (HS) degradation, has been linked to dysbiosis-associated rheumatoid arthritis [76] and, interestingly, a negative correlation between *Bacteroides plebeius* and omega-3 enriched diets was observed, which aligns with the anti-inflammatory properties of oily fish that can counteract the inflammation induced by this bacterium. Additionally, a low-carbohydrate diet has been shown to inhibit its growth [77].

Another bacterial species, *Roseburia faecis*, which plays a vital role in the breakdown of dietary polysaccharides to produce SCFAs like butyrate, shows a negative correlation with diets rich in animal proteins (such as oily fish, cheese, and ice cream) and low in carbohydrates [78]. The lack of suitable substrates in these foods, mainly carbohydrates and dietary fibers, is likely responsible for the negative correlation observed. Furthermore, *Roseburia faecis* thrives on fermenting sugars like fructose, glucose, maltose, cellobiose, raffinose, xylose, sorbitol, melibiose, amylopectin, and starch, while lactose (glucose + galactose), the primary sugar in milk, is not among its preferred fermentable sugars [79]. The Mediterranean diet, associated with the increased intestinal presence of Roseburia spp., has been found to promote the growth of this beneficial bacterial species [80]. Another species, Roseburia sp. 11SE39 also demonstrates a similar pattern. It is negatively correlated with diets high in animal protein and low in carbohydrates, which is consistent with the trend observed in our participants. This further supports the notion that diets rich in animal-derived foods like oily fish, cheese, and ice cream, which are abundant in animal fats and proteins and low in carbohydrates, can hinder the growth of this specific taxa, also emphasizing the influence of dietary composition on the abundance and activity of specific microbial species within the gut microbiota.

Another interesting observation was related to the presence and abundance of *Lactobacillus crispatus*, a dominant species in the cervicovaginal microbiome of Caucasian women, which has already been shown to be influenced by dietary factors. Specifically, studies have found that milk and dairy consumption promote a higher relative abundance of *Lactobacillus crispatus* in the vaginal microbiota due to its carbohydrate metabolism, particularly glycogen utilization in the vaginal environment [81]. Although this finding pertains to the mucosal environment of the vagina, it is worth noting that oral supplementation of *L. crispatus* may involve passage through the gut before reaching the final destination, thus allowing its detection in fecal samples (albeit transiently). Interestingly, a study proposed the concept of a probiotic cheese-based formula containing *L. crispatus* as a potential “gender probiotic food” for preventing gynecological infections [82]. This connection between increased intake of ice cream and Parmesan cheese and *L. crispatus* suggests a potential match with the literature. However, further investigation, including sex-based statistical correlations, could provide a better understanding of the relationship between *L. crispatus* and dietary factors.

The *Butyrate–producing bacterium SR1/5* is involved in carbohydrate metabolism, particularly the utilization of dietary fibers. Butyrate-producing bacteria are found in various classes within the Firmicutes phylum [69]. Butyrate, a short-chain fatty acid, is formed from sugar molecules through a series of reactions, ultimately leading to the liberation of butyrate from Butyryl-CoA. While the specific pathways utilized by SR1/5 are not well characterized, the majority of known butyrate-producing gut strains employ the butyryl-CoA:acetate CoA-transferase pathway [83]. In the context of the significant increase in Parmesan cheese, ice creams, and oily fish, it is essential to consider the sugar content in milk-derived products such as cheese and ice cream, as well as the biochemical pathways present in Firmicutes that facilitate the conversion of carbohydrates to butyrate. This connection between the *Butyrate–producing bacterium SR1/5* and the observed increase in milk-based foods provides a plausible link, suggesting that the consumption of these foods may influence the abundance or activity of SR1/5 in the gut.

The integration of microbiome and host-microbial metabolome analyses promises to illuminate the intricate metabolic interplay between the gut microbiota and the host in both health and disease. However, the elusive definition of a “healthy core gut microbiota gold standard” remains an ongoing challenge. While recognizing the absence of a universally accepted definition of a healthy microbial community structure, our study primarily aimed to comprehend the impact of dietary interventions on participants’ gut microbiota and their potential repercussions on overall health. Our approach to identifying beneficial and detrimental bacteria draws on established literature and empirical observations, considering broader taxonomic categories as indicators of potential microbial imbalances with functional significance. Rather than assuming that individual species or genus-level taxa would singularly transform microbiome function, we leveraged existing research to pinpoint specific bacterial taxa or patterns linked to various health outcomes or microbial community imbalances. These dietary recommendations targeted observed imbalances, striving to foster a more favorable gut microbial ecosystem. Furthermore, it is noteworthy that our study revealed substantial improvements in various physiological and anthropometric parameters, such as resting heart rate and BMI. These findings align with well-documented indicators of enhanced health status, underscoring the potential advantages of our dietary interventions for overall well-being.

In essence, our study seeks to deepen the comprehension of the intricate interplay between diet, gut microbiota, and host health. We aim to establish correlations between dietary interventions and changes in the microbiota and host responses, contributing to a broader understanding of personalized nutrition’s potential impact on health.

Nonetheless, achieving consensus in this realm remains challenging, especially given the substantial variation influenced by factors such as individual demographics, ethnicity, sex, lifestyle, diet, and age [84]. Gut bacteria release bioactive metabolites into the bloodstream, and advanced analytical tools like mass spectrometry and nuclear magnetic resonance enable the identification of disease-associated metabolites in various biological samples. This facilitates cooperative analyses of the microbiome, metabolome, and host phenotypes to unveil potential mechanistic links between the human and microbial ecosystems [85]. Future advances in microbiome-wide association studies, supported by bioinformatic algorithms and correlation coefficients, will enable further categorization of microbial genes into specific groups, such as metagenomic linkage groups, metagenomic species, co-abundance gene groups, or metagenomic species pan-genomes [86]. The resulting microbial gene catalog represents a rich data source to establish associations and predictions regarding health or disease status, leveraging the power of advanced machine learning technologies.

## 5. Conclusions

In conclusion, the study utilized a precision nutrition approach, integrating genetic profiles, microbiome composition, and physiological parameters to create personalized dietary plans. The proposed digitalized approach offers cost advantages through efficiency, scalability, and data analysis, as well as the benefits of personalization, real-time monitoring, continuous support, and behavior change, making it more advantageous compared to traditional methods. Its potential to revolutionize personalized nutrition interventions, offering individuals a more engaging, effective, and accessible way to optimize their dietary choices and overall health, combined with the obtained findings, highlights the importance of personalized nutrition in optimizing health and well-being, as well as the role of the gut microbiome in dietary interventions. Although these interesting results pave the way for the integration of nutritional approaches in the modulation of gut health, further research is needed to understand the underlying mechanisms and long-term implications.

## Figures and Tables

**Table 1:** **Categorization of genes analyzed.** The table provides a categorization of the several investigated genes. Each gene is listed along with its corresponding rs number, a unique identifier for genetic variations.

Super-Category	Category	Gene	rs_Number
Weight management	Carbohydrates	*ADRB2*	rs1042713
		*TCF7L2*	rs7903146
	Proteins	*FTO*	rs1558902
		*FTO*	rs9930506
		*FTO*	rs9939609
	Fats	*TCF7L2*	rs12255372
		*FTO*	rs9930506
		*PPM1K*	rs1440581
		*PPARG*	rs1801282
		*FTO*	rs9939609
	Snacking between meals	*MC4R*	rs17782313
	Sweet taste preference	*SLC2A2*	rs5400
	Biological clock	*CLOCK*	rs1801260
	Salt sensitivity	*ACE*	rs4343
		*AGT*	rs699
		*ATP2B1*	rs2681472
	Saturated fats	*APOE*	rs7412
		*APO3*	rs429358
		*APOA2*	rs5082
	ω6/ω3 fatty acids	*FADS1*	rs174546
		*FADS2*	rs174570
	Trans fats	*FADS1*	rs174546
		*LIPC*	rs1800588
		*APOC3*	rs5128
Sensitivities	Caffeine	*ADORA2A*	rs2298383
		*ADORA2A*	rs5751876
		*CYP1A2*	rs762551
	Alcohol	*ADH1C*	rs283411
		*GABRA2*	rs279858
	Lactose	*MCM6*	rs4988235
	Gluten	*HLA DQ 2.2*	rs2395182
		*HLA DQ 2.2*	rs4713586
		*HLA–DQA1*	rs2187668
		*HLA–DQB1*	rs7775228
		*HLA DQ*	rs7454108
Detoxification capacity and antioxidant needs	Detoxification capacity	*CYP1A2*	rs762551
		*GSTP1*	rs1695
	Antioxidant needs	*SOD2*	rs4880
		*CAT*	rs1001179
Vitamins	Vitamin A	*BCO1*	rs6564851
		*BCMO1*	rs7501331
	Vitamin B6	*ALPL*	rs4654748
	Vitamin B9—Folic and Folic acid	*MTHFR*	rs1801133
	Vitamin B12	*FUT2*	rs492602
		*TCN1*	rs526934
	Vitamin C	*SLC23A1*	rs10063949
		*SLC23A2*	rs6053005
	Vitamin D	*CYP2R1*	rs10741657
		*GC*	rs2282679
		*VDR*	rs2228570
	Vitamin E	*SCARB1*	rs11057830
		*TRIP6*	rs964184
Minerals	Low calcium levels	*CYP2R1*	rs2060793
		*GC*	rs7041
		*VDR*	rs2228570
	Increased calcium concentration	*CYP24A1*	rs1570669
	Low iron levels	*TMPRSS6*	rs4820268
		*TF*	rs1799852
		*TFR2*	rs7385804
	Iron overload	*HFE*	rs1799945
	Magnesium	*MUC1*	rs4072037
Sports profile	Endurance	*ACE*	rs4343
		*PPARA*	rs4253778
		*HFE*	rs1799945
		*NFIA–AS2*	rs1572312
		*ADRB3*	rs4994
		*HIF1A*	rs11549465
		*PPARD*	rs2016520
		*NRF2*	rs7181866
	Strength	*MSTN*	rs1805086
		*PPARA*	rs4253778
		*ACTN3*	rs1815739
		*AGT*	rs699
	Power	*ACTN3*	rs1815739
		*NOS3*	rs2070744
		*ACE*	rs4343
		*AGT*	rs699
		*ADRB2*	rs1042713
	Aerobic capacity (VO_2_ max)	*ADRB2*	rs1042713
		*CRP*	rs1205
		*GSTP1*	rs1695
		*ACE*	rs4343
	Muscle mass hypertrophy	*LEPR*	rs1137101
	Motivation to exercise	*BDNF*	rs6265
		*COMT*	rs4680
Injury predisposition	Pain tolerance	*COMT*	rs4680
	Jumper’s knee and tennis elbow injuries	*COL5A1*	rs12722
		*COL1A1*	rs1800012
		*COL3A1*	rs1800255
	Achille’s tendon injury	*COL5A1*	rs12722
	Musculoskeletal health	*BTNL2*	rs10947262
		*SPTBN1*	rs11898505
	Exercise rehabilitation	*CRP*	rs1205
		*SOD2*	rs4880
		*ACTN3*	rs1815739

**Table 2:** **Subject characteristics and macronutrient composition of different dietary types.** The table provides an overview of the subjects’ characteristics, their % of macronutrient intake, and the macronutrient composition of the proposed dietary types. The subjects are identified by unique codes (e.g., WL010114) and their corresponding age. The table presents the specific diet type followed by each subject, including Mediterranean and Ketogenic/Low Carb, along with the percentage distribution of macronutrients. The macronutrient breakdown includes carbohydrates (CHO), protein (PRO), and lipids (LIP) expressed as percentages of the whole macronutrient intake. The data highlight variations in macronutrient proportions among different dietary types, providing insights into the dietary patterns followed by the subjects in the study.

Subject	Age	% Macronutrient Intake	Diet Type	% Macronutrients Diet-Provided
WL010114	26	CHO: 62.8%—PRO: 18.3%—LIP: 18.9%	Mediterranean	CHO: 47.9%—PRO: 21.9%—LIP: 30.2%
WL010112	28	CHO: 61.9%—PRO: 18.0%—LIP: 20.1%	Mediterranean (with high fish intake)	CHO: 47.1%—PRO: 23.3%—LIP: 29.5%
WL010111	44	CHO: 64.2%—PRO: 19.0%—LIP: 16.8%	Mediterranean (with high fish intake)	CHO: 45.2%—PRO: 24.4%—LIP: 30.4%
WL010107	46	CHO: 52.6%—PRO: 27.5%—LIP: 19.8%	Mediterranean	CHO: 47.5%—PRO: 23.0%—LIP: 29.5%
WL010106	52	CHO: 67.5%—PRO: 20.0%—LIP: 12.5%	Mediterranean	CHO: 44.0%—PRO: 21.7%—LIP: 34.3%
WL010105	50	CHO: 62.2%—PRO: 22.1%—LIP: 15.7%	Mediterranean (with high fish intake)	CHO: 42.2%—PRO: 22.8%—LIP: 34.9%
WL010108	40	CHO: 61.2%—PRO: 20.5%—LIP: 18.3%	Ketogenic/Low Carb	CHO: 36.9%—PRO: 33.3%—LIP: 29.8%

**Table 3:** **Changes in food items between T_CTRL_ and T_DIET_.** Comparison of mean values and statistical analysis for various food items consumed in T_CTRL_ (baseline) and T_DIET_ (follow-up) stages of the study. The t-statistics, trend (increase or decrease), and FDR-corrected *p*-values are provided to indicate the significance of the observed changes. (*) indicates statistical significance at *p* ≤ 0.05.

Food Item	T_CTRL_ Mean ± SD	T_DIET_ Mean ± SD	t-Statistics	Trend	*p*-Value
Cereal Bars (g)	13.1 ± 13.0	0.0 ± 0.0	2.673	Decrease	0.05 (*)
Chocolate (g)	18.7 ± 18.8	0.6 ± 1.7	2.466	Decrease	0.05 (*)
Ice Cream (g)	16.8 ± 23.1	60.7 ± 34.4	−2.796	Increase	0.05 (*)
Parmesan Cheese (g)	17.7 ± 4.4	24.2 ± 6.1	−3.113	Increase	0.05 (*)
Oily Fish (g)	109.4 ± 93.5	196.5 ± 131.5	−3.672	Increase	0.05 (*)

**Table 4:** **Changes in nutritional variables before and after the intervention.** This table presents the mean values and standard deviations (±SD) of various nutritional variables, measured at two time points, T_CTRL_ (baseline) and T_DIET_ (follow-up), along with the t-statistics, trend, and FDR-corrected *p*-values. The data show the changes in calorie intake, macronutrient composition, and selected micronutrients following the intervention. The asterisks (*) indicate statistically significant changes (*p* ≤ 0.05).

Variable	T_CTRL_ Mean ± SD	T_DIET_ Mean ± SD	t-Statistics	Trend	*p*-Value
Intake (Kcal)	1457 ± 554	1488 ± 549	−0.580	Increase	0.583
Carbohydrates (%)	57.1 ± 5.1	51.7 ± 11.1	1.445	Decrease	0.198
Proteins (%)	20.8 ± 3.3	25.5 ± 8.5	−1.380	Increase	0.217
Fibers (%)	4.7 ± 1.5	4.9 ± 1.5	−0.633	Increase	0.550
Calcium (mg)	413 ± 121	601 ± 251	−2.732	Increase	0.05 (*)
Potassium (mg)	1476 ± 712	1815 ± 615	−2.693	Increase	0.05 (*)
Phosphorus (mg)	572 ± 241	709 ± 292	−2.556	Increase	0.05 (*)
Sodium (mg)	1200 ± 717	1388 ± 760	−3.027	Increase	0.05 (*)
Zinc (mg)	4 ± 2	5 ± 2	−2.565	Increase	0.05 (*)

**Table 5:** **Changes in anthropometric and physiological parameters over time.** This table presents the mean values and standard deviations (SD) of various anthropometric and physiological parameters for the study participants at T_CTRL_ (baseline) and T_DIET_ (follow-up). The anthropometric variables include weight (kg), BMI (kg/m2), basal metabolism (kcal), physical activity (kcal), body fat (%), muscle mass (kg), bone mass (kg), and water content (%). The physiological variables include resting heart rate (bpm), average heart rate (bpm), duration of deep sleep (min), duration of shallow sleep (min), and duration of REM sleep (min). The t-statistics, trend direction, and FDR-corrected *p*-values are also provided. Significant changes are denoted by asterisks (*) for *p*-value ≤ 0.05.

Group	Variable	T_CTRL_ Mean ± SD	T_DIET_ Mean ± SD	t-Statistics	Trend	*p*-Value
Anthropometric	Weight (kg)	69.1 ± 12.6	67.2 ± 11.5	2.116	Decrease	0.08
	BMI (kg/m^2^)	23.1 ± 2.8	22.4 ± 2.2	2.343	Decrease	0.05 (*)
	Basal Metabolism (kcal)	1327 ± 279	1317 ± 249	0.486	Decrease	0.64
	Physical Activity (kcal)	402 ± 254	441 ± 280	−0.804	Increase	0.45
	Body Fat (%)	27.1 ± 7.2	26.2 ± 6.3	1.604	Decrease	0.16
	Muscle (kg)	47.1 ± 10.8	47.1 ± 10.0	−0.001	Increase	0.10
	Bone Mass (kg)	2.7 ± 0.4	2.7 ± 0.4	0.427	Decrease	0.68
	Water (%)	50.0 ± 3.9	51.4 ± 3.5	−2.300	Increase	0.06
Physiological	Resting Heart Rate (bpm)	60.9 ± 7.1	57.9 ± 7.3	2.571	Decrease	0.05 (*)
	Average Heart Rate (bpm)	73.8 ± 4.5	73.1 ± 5.0	1.033	Decrease	0.34
	Deep Sleep (min)	85.3 ± 12.3	88.0 ± 8.2	−0.685	Increase	0.52
	Shallow Sleep (min)	281.5 ± 20.8	261.2 ± 14.4	3.682	Decrease	0.05 (*)
	REM (min)	59.5 ± 15.2	58.7 ± 17.1	0.252	Increase	0.81

**Table 6:** **Changes in diversity and microbial abundance in response to intervention.** This table presents the mean values (T_CTRL_ and T_DIET_) and statistical analysis results for several variables related to diversity and microbial abundance in terms of both phylum and species, for which only those exhibiting statistically significant variations were included. The t-statistics, trend, and FDR-corrected *p*-values are provided to examine the changes observed between the two time points. (*) stands for *p*-value ≤ 0.05; (°) stands for *p*-value ≤ 0.1.

Group	Variable	T_CTRL_ Mean ± SD	T_DIET_ Mean ± SD	t-Statistics	Trend	*p*-Value
Diversity	Richness	409.7 ± 44.8	528.1 ± 110.9	−4.355	Increase	0.05 (*)
	Pielou’s evenness	0.627 ± 0.026	0.634 ± 0.029	−0.626	Increase	0.55
	Shannon diversity	3.76 ± 0.12	3.96 ± 0.11	−3.396	Increase	0.05 (*)
Phyla	Firmicutes	0.54 ± 0.14	0.60 ± 0.15	−1.895	Increase	0.11
	Bacteroidetes	0.27 ± 0.20	0.21 ± 0.19	0.837	Decrease	0.43
	Proteobacteria	0.08 ± 0.14	0.06 ± 0.05	0.682	Decrease	0.52
	Actinobacteria	0.07 ± 0.10	0.08 ± 0.07	−0.283	Increase	0.79
	Verrucomicrobia	0.02 ± 0.04	0.04 ± 0.05	−0.896	Increase	0.40
Species	*Acinetobacter junii*	(1.2 ± 1.6) × 10^−5^	(2.8 ± 2.0) × 10^−5^	−2.525	Increase	0.05 (*)
	*Alistipes finegoldii*	(2.9 ± 3.1) × 10^−4^	(1.3 ± 2.0) × 10^−4^	2.662	Decrease	0.05 (*)
	*Alistipes finegoldii DSM 17242*	(2.4 ± 4.5) × 10^−6^	(6.9 ± 6.8) × 10^−6^	−2.602	Increase	0.05 (*)
	*Bacteroides plebeius*	(5.6 ± 5.5) × 10^−5^	(6.9 ± 6.8) × 10^−5^	3.046	Decrease	0.05 (*)
	*Klebsiella* sp.	0.0 ± 0.0	(1.5 ± 1.7) × 10^−5^	−2.367	Increase	0.05 (*)
	*Klebsiella* sp. *8.1T*	0.0 ± 0.0	(1.2 ± 1.3) × 10^−5^	−2.482	Increase	0.05 (*)
	*Klebsiella* sp. *XW111*	(7.6 ± 2.0) × 10^−7^	(7.8 ± 7.7) × 10^−5^	−2.651	Increase	0.05 (*)
	*Klebsiella* sp. *YSI6A*	0.0 ± 0.0	(2.9 ± 3.2) × 10^−5^	−2.445	Increase	0.05 (*)
	*Klebsiella variicola*	0.0 ± 0.0	(8.7 ± 9.9) × 10^−5^	−2.318	Increase	0.06 (°)
	*Lachnospiraceae bacterium DJF RP14*	(2.7 ± 5.7) × 10^−4^	(3.9 ± 6.2) × 10^−4^	−2.407	Increase	0.05 (*)
	*Lachnospiraceae bacterium DJF VP18k1*	(1.5 ± 1.8) × 10^−4^	(3.6 ± 3.2) × 10^−4^	−2.768	Increase	0.05 (*)
	*Lactobacillus crispatus*	(4.2 ± 3.1) × 10^−5^	(1.7 ± 1.6) × 10^−4^	−2.467	Increase	0.05 (*)
	*Roseburia faecis*	(1.2 ± 1.2) × 10^−4^	(2.0 ± 3.4) × 10^−5^	2.587	Decrease	0.05 (*)
	*Roseburia* sp. *11SE39*	(1.5 ± 1.4) × 10^−4^	(2.1 ± 3.3) × 10^−4^	2.748	Decrease	0.05 (*)
	*Bacterium NLAE–zl–P167*	(1.2 ± 1.2) × 10^−5^	(1.6 ± 4.1) × 10^−6^	2.468	Decrease	0.05 (*)
	*Butyrate–producing bacterium PH07BW10*	(2.5 ± 2.6) × 10^−3^	(1.3 ± 2.0) × 10^−3^	2.385	Decrease	0.05 (*)
	*Butyrate–producing bacterium SR1/5*	(4.0 ± 3.8) × 10^−4^	(8.7 ± 5.9) × 10^−4^	−2.597	Increase	0.05 (*)

## Data Availability

Data are available upon request to the corresponding author.

## Supplementary Materials

The following supporting information can be downloaded at https://www.mdpi.com/article/10.3390/nu15183931/s1, Section S1: Evaluation of the stability and reliability of the individual’s gut microbiome before the nutritional intervention; Section S2: Personalized Interventions and Comprehensive Evaluations for Individualized Health Optimization. Section S3: Evaluation of the inter-individual variations. Figure S1. Beta diversity analysis between T1 and T2. Beta diversity was investigated using *Bray–Curtis* distance and represented by Principal coordinates analysis (PCoA). Statistical differences between the two groups (T1 and T2) were assessed by Permutational Multivariate Analysis of Variance (PERMANOVA) test (*p* > 0.05). Different shapes represent different time points, while different colors are associated with the various studied samples; Figure S2. Relative abundance at genus level. Relative abundance at genus level was investigated on the 20 most representative genera. Each line links samples belonging to the same individual, whereas box plot chart is representative of the median and 25th and 75th percentiles (T_CTRL_: dark green, T_DIET_: dark red). Statistical differences were inferred using Wilcoxon test for paired samples followed by Bonferroni’s correction. Table S1. Results of the PERMANOVA test on beta-diversity before and after the nutritional intervention. This table presents the results of R^2^ and *p*-value of the Permutational Multivariate Analysis of Variance (PERMANOVA) test performed on T1, T2, and T3, respectively, to evaluate the stability of the microbial composition before the nutritional intervention. Table S2. Personalized intervention and evaluations for different subjects. This table provides an overview of individual subjects along with their age, physiological evaluation, genome evaluation, microbiome evaluation, and principal intervention. The subjects’ energy requirements, specific sensitivities, genetic predispositions, and microbiome composition are highlighted. Personalized interventions, including dietary modifications and supplementation recommendations, are tailored based on each subject’s characteristics. The interventions aim to address imbalances in the microbiome, optimize nutrient intake, and promote overall health and well-being.
