# Supplementary material for: Unraveling the Gut Microbiome–Diet Connection: Exploring the Impact of Digital Precision and Personalized Nutrition on Microbiota Composition and Host Physiology
Source: Nutrients. 2023 Sep 11;15(18):3931. doi: 10.3390/nu15183931 (PMC10537332; doi:10.3390/nu15183931)
Supplement: Supplementary file 1 [file nutrients-15-03931-s001.zip › nutrients-2508150-supplementary.pdf]

nutrients

MDPI

Supplementary Materials

Unraveling the Gut Microbiome-Diet Connection: Exploring the Impact of Digital Precision and Personalized Nutrition on Microbiota Composition and Host Physiology

Giada Bianchetti [1,2,†], Flavio De Maio [3,†], Alessio Abeltino [1,2], Cassandra Serantoni [1,2], Alessia Riente [1,2], Giulia Santarelli [3,4], Maurizio Sanguinetti [3,4], Giovanni Delogu [4,5], Roberta Martinoli [6], Silvia Barbaresi [7], Marco De Spirito [1,2] and Giuseppe Maulucci [1,2,*]

1 Department of Neuroscience, Biophysics Sections, Università Cattolica del Sacro Cuore, Largo Francesco Vito, 1, 00168 Rome, Italy; giada.bianchetti@unicatt.it (G.B.); alessio.abeltino@unicatt.it (A.A.); cassandra.serantoni@unicatt.it (C.S.); alessia.riente@unicatt.it (A.R.); marco.despirito@unicatt.it (M.D.S.)
2 Fondazione Policlinico Universitario "A. Gemelli" IRCCS, 00168 Rome, Italy
3 Dipartimento di Scienze di Laboratorio e Infettivologiche, Fondazione Policlinico Universitario "A. Gemelli", IRCCS, 00168 Rome, Italy; flavio.demaio@unicatt.it (F.D.M.); giulia.santarelli@unicatt.it (G.S.); maurizio.sanguinetti@unicatt.it (M.S.)
4 Dipartimento di Scienze Biotecnologiche di Base, Cliniche Intensivologiche e Perioperatorie—Sezione di Microbiologia, Università Cattolica del Sacro Cuore, 00168 Rome, Italy; giovanni.delogu@unicatt.it
5 Mater Olbia Hospital, 07026 Olbia, Italy
6 Società Italiana di Medicina Estetica, 00195 Rome, Italy; dott.roberta.martinoli@gmail.com
7 Faculty of Medicine and Health Sciences—Department of Movement and Sports Sciences-Watersportlaan 2, Ghent University, Ghent, Belgium; silvia.barbaresi@ugent.be
* Correspondence: giuseppe.maulucci@unicatt.it; Tel.: +39-06-3015-4265
† These authors contributed equally to this work.

S1. Evaluation of the stability and reliability of the individual's gut microbiome before the nutritional intervention

To evaluate the stability and reliability of the individual's gut microbiome before the nutritional intervention, the beta diversity, a measure reflecting the similarity or dissimilarity of the two microbiome sampling used as a control reference, namely T1 (April 2022) and T2 (May 2022), was evaluated using *Bray-Curtis* distance and represented by Principal coordinates analysis (PCoA) in the following Figure S1.

Looking at the graph in Figure S1, which represents on the x and y axis the two principal components, it is evident that the two points T1 and T2 (circle and square, respectively) are overlapping for each sample, indicating no variation in the microbiota. Statistical differences between the groups were assessed by Permutational Multivariate Analysis of Variance (PERMANOVA) test, which relies on a distribution of data, and whose results, including also the values at T3, are reported in the following Table S1.

Table S1. Results of the PERMANOVA test on beta-diversity before and after the nutritional intervention. This table presents the results of R^2 and p-value of the Permutational Multivariate Analysis of Variance (PERMANOVA) test performed on T1, T2, and T3, respectively, to evaluate the stability of the microbial composition before the nutritional intervention.

Timepoints	R^2	*p*-Value
T1 vs T2	0.200	0.922
T1 vs T3	0.053	0.668
T2 vs T3	0.038	0.834

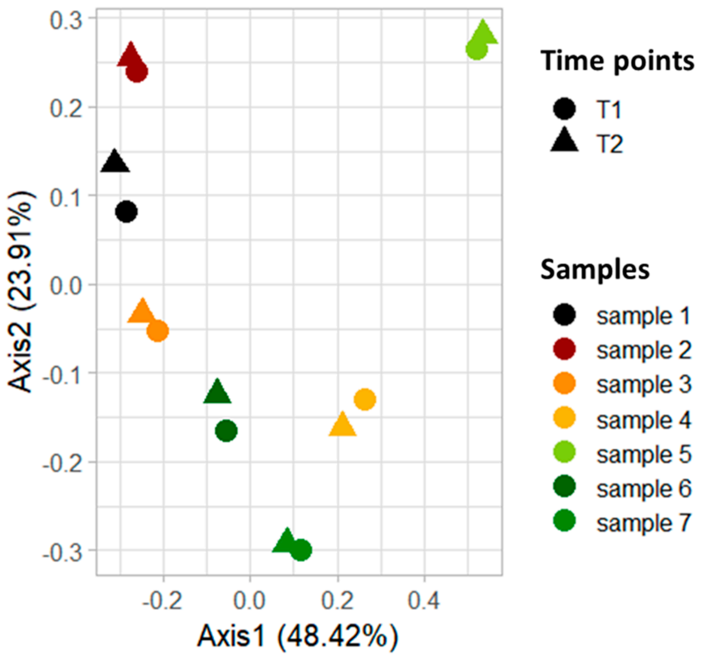

Figure S1. Beta diversity analysis between T1 and T2. Beta diversity was investigated using *Bray-Curtis* distance and represented by Principal coordinates analysis (PCoA). Statistical differences between the two groups (T1 and T2) were assessed by Permutational Multivariate Analysis of Variance (PERMANOVA) test ($p > 0.05$). Different shapes represent different time points, while different colors are associated with the various studied samples.

S2. Personalized Interventions and Comprehensive Evaluations for Individualized Health Optimization

The following Table S2 showcases a comprehensive compilation of personalized interventions and evaluations, carefully tailored by nutritionists using a combination of analyses and data obtained from various devices. By integrating physiological, genome, and microbiome evaluation, they have developed personalized interventions for each subject, taking into account factors such as age, anthropometric and physiological characteristics, genetic predispositions, and microbiome composition, to address energy requirements, nutrient sensitivities, and microbial community imbalances on an individual level. This personalized evaluation, especially regarding microbiome composition, was performed by the nutritionists based on their expertise and available literature [40-44], in order to define our “gold standard”, which was used to evaluate the deviations of the microbial communities and as a guide in the interpretation of the gut microbiota tests and action-taking on dietary plans. Participants were advised to modify their dietary choices on a daily basis, through the suggestion of a personalized menu which included 3 main meals (breakfast, lunch, and dinner) and two snacks. In addition, alternatives to each food were provided for each meal, allowing for the maintenance of the nutrient balance. Suggestions regarding the physical activity were also provided to subjects.

This pioneering approach highlights the crucial interplay between an individual's genetics, physiology, and gut microbiome, laying the foundation for targeted and personalized dietary interventions supported by comprehensive evaluations.

Table S2. Personalized intervention and evaluations for different subjects. This table provides an overview of individual subjects along with their age, physiological evaluation, genome evaluation, microbiome evaluation, and principal intervention. The subjects' energy requirements, specific sensitivities, genetic predispositions, and microbiome composition are highlighted. Personalized interventions, including dietary modifications and supplementation recommendations, are tailored based on each subject's characteristics. The interventions aim to address imbalances in the microbiome, optimize nutrient intake, and promote overall health and well-being.

Subject	Age	Physiological Evaluation	Genome Evaluation	Microbiome Evaluation	Principal Intervention
WL010114	26	Energy requirement of 1900 kcal for weight loss. Frequent episodes of diarrhea. Probiotics and anti-acid medication.	Heightened sensitivity to carbohydrates. Predisposed to obesity and type 2 diabetes, with a preference for sweet foods. High cholesterol levels. Increased sensitivity to gluten and alcohol. Antioxidants and various vitamins need to be supplemented. Greater affinity for endurance sports and strength training.	Imbalance in the bacterial composition. Deficiency of Bacteroidetes and excess of Proteobacteria (small intestinal bacterial overgrowth (SIBO) or inflammatory bowel disease (IBD)). Faecalibacterium is low and needs to be increased. Akkermansia is slightly elevated (IBD?).	Increase intake of protein-rich foods. Reduce intake of trans fats. Incorporate omega-3 fatty acids through fatty fish and plant-based sources. Limit salt intake by avoiding consuming processed or industrial foods.
WL010112	28	Estimated energy requirement: 1800 kcal/day.	Greater sensitivity to carbohydrate and lipid, while proteins are better tolerated. Be cautious with alcohol and gluten. Requires high doses of antioxidants, vitamin A, B9 (folate), vitamin C, vitamin E, and calcium. Predisposition for aerobic activities.	High presence of Proteobacteria and high Gram-negativity. Presence of dysbiotic Lachnospiraceae (Ruminococcus) indicating increased abdominal fermentation (SIBO?). Low abundance of Akkermansia and Prevotella, as well as of Faecalibacterium, that need to be increased.	A low-histamine diet is recommended. Incorporate specific foods and supplements such as berberine, curcumin, kefir, and other fermented foods. Adjuste protein and fat sources, favoring vegetables, legumes, and whole grains.
WL010111	44	Set the diet to approximately 2000-2300 kcal. Recent occurrence of gastrointestinal episodes.	Genetic predisposition to obesity, diabetes, and metabolic diseases with a high-carbohydrate diet, but better metabolic response to a high-protein diet. Favorable genetic predisposition for VO_{2max} (maximal oxygen consumption at the muscle level) and efficient mitochondrial activity.	Suspected fermentative dysbiosis with an excess of Firmicutes. Lower values of Akkermansia and Faecalibacterium than the average. Low abundance of Prevotella.	Adjust the diet to rebalance the presence of Firmicutes and Bacteroidetes by reducing carbohydrates and fats, increasing vegetable and protein intake, and opting for whole grains and gluten-free options. Alcohol consumption should be reduced, and supplementation with

			Genetic predisposition for strength, power, and speed activities.		berberine, kefir, and Bifidobacterium longum is recommended.<br>Increasing fiber, vegetables, and legumes is necessary to promote the growth of Prevotella.
WL010107	46	Calories intake: 1700 kcal/day.<br>Elevated waist and hip circumferences.<br>Low HDL.	High predisposition to predispose metabolic diseases; a diet rich in proteins is better tolerated.<br>High sensitivity to alcohol and gluten.<br>Greater predisposition towards endurance sports and strength training.	Significant fermentative dysbiosis, with an excess of Faecalibacterium.<br>Too high Firmicutes and Actinobacteria, while Bacteroidetes and Proteobacteria are too low.<br>Prevotella and Akkermansia are low.	Moderate consumption of trans and saturated fats.<br>Supplement the diet with antioxidants and vitamins.<br>Reduce the consumption of sugars and sweets in the diet and fats in favor of lean proteins.<br>Consume whole grains with a low glycemic index.<br>Necessary to increase carbonates in the lumen, through vegetables and fruits.<br>Berberine, Quassia amara, Juglans regia, or fasting can help increasing levels of Akkermansia.<br>Consumption of rice and plant-based food is recommended.
WL010106	52	Calories requirement: 2300 kcal/day.	High sensitivity to fats in the diet, especially trans fats.<br>High sensitivity to caffeine, alcohol, and gluten.<br>Excellent genetic predisposition for endurance sports.	Proteobacteria and Actinobacteria are much higher than the average control values.<br>High presence of Collinsella.<br>Faecalibacterium and Prevotella are low.<br>High levels of Bifidobacteria.<br>Slightly low values of Akkermansia.	Better distribute the calories between the two snacks.<br>Reduce the consumption of processed, refined, and packaged foods, as well as red meats and dairy products, in favor of a more vegetarian diet rich in gluten-free whole grains and legumes.
WL010105	50	Hypocaloric requirement: 900 kcal for weight loss.<br>Steeps little and moves little.	High sensitivity to carbohydrates and fats, greater affinity for a protein diet.<br>High sensitivity to caffeine, alcohol, and gluten.	Presence of mild fermentative dysbiosis.<br>High biodiversity.	A change in the diet is recommended, reducing the consumption of sweets, refined sugars, and refined foods, and

		High abdominal and hip circumference.		Excess of Firmicutes and deficiency of Bacteroidetes; high levels of Proteobacteria and Actinobacteria.<br>Excess of Bacteroides and Faecalibacterium.<br>Very low levels of Prevotella.<br>Excess of Blautia.<br>Low abundance of Akkermansia and high Bifidobacterium.	increasing the intake of whole grains, legumes, and vegetables.<br>Prefer the consumption of plant proteins over animal proteins.<br>Berberine, kefir, or other fermented foods, and a dietary regimen with intermittent fasting is recommended.<br>Reduce the proportion of saturated fats in favor of omega-3s.<br>Prefer small fish due to difficulty in eliminating heavy metals (such as mercury).<br>Reduce the amount of added salt.
WL010108	40	Estimated daily hypocaloric requirement: 1000 kcal/day.	High sensitivity to carbohydrates, with a risk of type 2 diabetes. Proteins are better tolerated.<br>High sensitivity to fats, especially saturated and trans fats.<br>High sensitivity to caffeine, alcohol, and gluten.<br>Predisposition towards strength and power physical activities, less for aerobic activities.	Excessive presence of Firmicutes and Actinobacteria.<br>Low levels of Proteobacteria.<br>Extreme deficiency of Bacteroidetes.<br>Fermentative dysbiosis, higher risk of diabetes, and suspicion of SIBO.<br>Faecalibacterium and Prevotella are low.	Need for a higher intake of antioxidants, vitamins A, C, E, B6, B9, B12, calcium, and iron.<br>A gluten-free, low FODMAPS diet is recommended to reduce the levels of Bifidobacteria and Firmicutes in favor of Bacteroidetes.<br>To increase Faecalibacterium growth, curcumin, Quassia amara, or Juglans Regia are recommended.<br>A diet rich in vegetables and fruits is required to increase Gram negativity, while a diet rich in proteins and fats to increase Bacteroidetes.

S3. Evaluation of the inter-individuals' variations

To evaluate inter-individuals' variations, relative abundance at genus level was investigated on the 20 most representative genera, and results are presented in the following Figure S2.

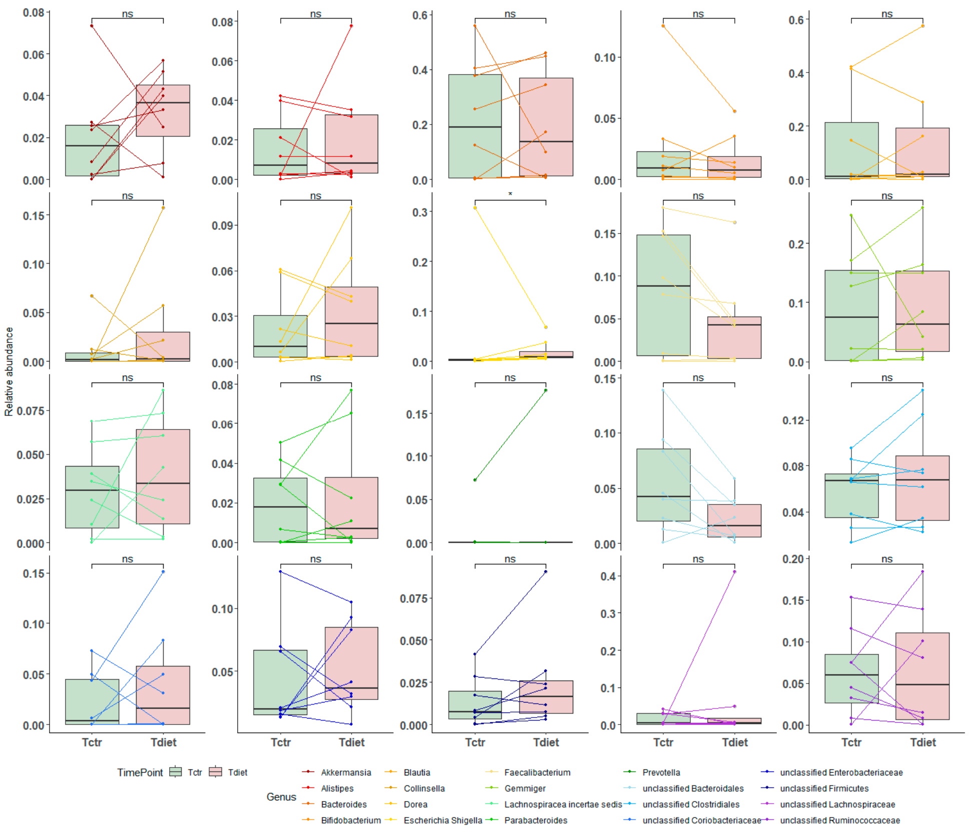

Figure S2. Relative abundance at genus level. Relative abundance at genus level was investigated on the 20 most representative genera. Each line links samples belonging to the same individual, whereas box plot chart is representative of the median and 25th and 75th percentiles (T_{CTRL}: dark green, T_{DIET}: dark red). Statistical differences were inferred using Wilcoxon test for paired samples followed by Bonferroni's correction.

In Figure S2, each line links samples belonging to the same individual, whereas box plot chart is representative of the median and 25th and 75th percentiles (T_{CTRL}: dark green, T_{DIET}: dark red). Statistical differences were inferred using Wilcoxon test for paired samples followed by Bonferroni's correction. Looking at these graphs, it is possible to observe limited statistical significance, which is reasonable given the nature of the analysis. Indeed, as our main focus is to investigate potential changes between the different time points rather than comparing distinct groups, it is not expected that changes will consistently occur in the same direction. Therefore, while the findings may not exhibit strong significance, they are in line with our research objective of assessing variations across time points. Furthermore, these results underscore the importance of a personalized longitudinal study, as it highlights variations at an individual level. Even within the same community, there can be substantial differences in the microbiota composition among individuals. This emphasizes the significance of considering personalized factors and characteristics when interpreting results in the context of gut microbiota analysis.
